# Tools for surveillance of anti-malarial drug resistance: an assessment of the current landscape

**DOI:** 10.1186/s12936-018-2185-9

**Published:** 2018-02-08

**Authors:** Christian Nsanzabana, Djibrine Djalle, Philippe J. Guérin, Didier Ménard, Iveth J. González

**Affiliations:** 1 Foundation for Innovative New Diagnostics (FIND), Geneva, Switzerland; 2 WorldWide Antimalarial Resistance Network, Oxford, UK; 3 Centre for Tropical Medicine and Global Health, Nuffield Department of Medicine, University of Oxford, Oxford, UK; 4 Unité Biologie des Interactions Hôte-Parasite, Institut Pasteur, Paris, France

**Keywords:** *Plasmodium falciparum*, Antimalarial, Resistance, Drug, Molecular, Markers, Surveillance, Landscape

## Abstract

To limit the spread and impact of anti-malarial drug resistance and react accordingly, surveillance systems able to detect and track in real-time its emergence and spread need to be strengthened or in some places established. Currently, surveillance of anti-malarial drug resistance is done by any of three approaches: (1) in vivo studies to assess the efficacy of drugs in patients; (2) in vitro/ex vivo studies to evaluate parasite susceptibility to the drugs; and/or (3) molecular assays to detect validated gene mutations and/or gene copy number changes that are associated with drug resistance. These methods are complementary, as they evaluate different aspects of resistance; however, standardization of methods, especially for in vitro/ex vivo and molecular techniques, is lacking. The World Health Organization has developed a standard protocol for evaluating the efficacy of anti-malarial drugs, which is used by National Malaria Control Programmes to conduct their therapeutic efficacy studies. Regional networks, such as the East African Network for Monitoring Antimalarial Treatment and the Amazon Network for the Surveillance of Antimalarial Drug Resistance, have been set up to strengthen regional capacities for monitoring anti-malarial drug resistance. The Worldwide Antimalarial Resistance Network has been established to collate and provide global spatial and temporal trends information on the efficacy of anti-malarial drugs and resistance. While exchange of information across endemic countries is essential for monitoring anti-malarial resistance, sustainable funding for the surveillance and networking activities remains challenging. The technology landscape for molecular assays is progressing quite rapidly, and easy-to-use and affordable new techniques are becoming available. They also offer the advantage of high throughput analysis from a simple blood spots obtained from a finger prick. New technologies combined with the strengthening of national reference laboratories in malaria-endemic countries through standardized protocols and training plus the availability of a proficiency testing programme, would contribute to the improvement and sustainability of anti-malarial resistance surveillance networks worldwide.

**Electronic supplementary material:**

The online version of this article (10.1186/s12936-018-2185-9) contains supplementary material, which is available to authorized users.

## Background

“Anti-malarial drug resistance” is defined as the “ability of a *Plasmodium* parasite strain to survive and/or to multiply despite the administration and absorption of a drug given in doses equal to or higher than the recommended ones, but within tolerance of the human subject” [1, 2]. Anti-malarial drug resistance is a global threat to malaria control and elimination, and is of particular concern in *Plasmodium falciparum*, the deadliest species that infects human. Resistance to anti-malarial drugs has also been reported in *Plasmodium vivax*; even if its extent is of lesser magnitude compared to *P. falciparum*, it is also becoming an increasing concern in vivax endemic regions, and a serious public health problem [3–5].

The first synthesized drug to become widely used to treat malaria was chloroquine (CQ). CQ was introduced in the early 1940s, however *P. falciparum* resistant parasites emerged by the early 1960s [6, 7]. Population genetic studies using molecular markers and microsatellites showed that *P. falciparum* CQ resistance (*Pf*CQR) emerged simultaneously in different geographical regions [8, 9]. There were at least four different origins for *Pf*CQR, one in Southeast Asia on the Thai-Cambodia border, a second in Papua New Guinea, a third in Colombia and another one in Venezuela [9]. *Plasmodium vivax* resistance to CQ (*Pv*CQR) emerged in the late 1980s in Papua New Guinea and Indonesia, and has been observed later in other regions, but its extent so far is still difficult to assess [4, 10]. CQ is still recommended to treat *P. vivax* infections in most endemic settings [3].

After *Pf*CQR spread throughout most of malaria-endemic countries, the drug was replaced by sulfadoxine-pyrimethamine (SP) as first-line therapy. However, resistance to SP developed rapidly, appearing first in Southeast Asia within 1 year of its introduction [11, 12]. Subsequently, low levels of resistance to SP emerged in different places in Southeast Asia, Africa and Latin America [13–16], but the highly resistant parasites followed the same path than *Pf*CQR, spreading from Southeast Asia to Africa [17]. After SP failure, mefloquine (MQ) was then used to replace SP; predictably, resistance developed within 5 years of its widespread use [18, 19]. Following the failure of MQ in Southeast Asia, the use of drugs in combination was, therefore, proposed as the best way to circumvent resistance, as the probability of a parasite to develop resistance to two drugs simultaneously is considered to be lower than for a single drug [20, 21].

Artemisinin derivatives were discovered in the 1970s, and are the most potent anti-malarial drugs available to date, showing rapid and steep declines in parasite density, despite their short half-life [20]. When combined with drugs that have longer half-life, the partner drug can clear the few remaining parasites, making the combination treatment highly efficacious, and less prone to resistance [21, 22]. Artemisinin-based combination therapy (ACT) is now the mainstay for malaria treatment in endemic regions, following recommendations from the World Health Organization (WHO) [23]; and initially proved to be highly efficacious in all endemic countries during the last 15 years [24–26]. However, parasites with decreased susceptibility to artemisinin derivatives have been reported from Southeast Asia [27–29], as well as resistance to the partner drug in the same region [30–32].

Artemisinin resistance has been associated with several point mutations in a propeller domain of a *kelch* gene located on the chromosome 13 [33, 34], however the exact mechanism of resistance is not yet fully understood, and so far those mutations associated with artemisinin resistance have not been detected outside of the Greater sub-Mekong region, China and Guyana [35, 36]. Multiple foci of origin for those mutations have already been discovered [35–37], and resistance may emerge in different regions with different epidemiological backgrounds [38]. To date, there is no alternative to ACTs, and the next generation of anti-malarial drugs will not be available on the market before several years [39, 40].

Nowadays, surveillance of anti-malarial drug resistance relies on three different and complementary approaches: in vivo efficacy studies for the detection of treatment failures, in vitro assessment of parasite sensitivity to drugs, and the detection of molecular signatures in the parasite associated with drug resistance. These different approaches have their own advantages and challenges. The in vivo assessments provides information about the efficacy of the studied drug in patients, but are difficult to conduct because of the heavy logistics and cost. This approach is particularly challenging in low transmission areas where thousands of patients need to be screened. Moreover, treatment outcomes are confounded by many other factors, such as acquired immunity, treatment adherence, nutrition status, pregnancy and pharmacogenetics [41]. In vitro methods provide useful information on the parasite susceptibility, but require substantial laboratory infrastructure and highly trained staff. Validated molecular markers are highly relevant to detect and monitor in real time the geospatial distribution of resistant parasites and their prevalence in a parasite population is often a good indicator of the level of clinical resistance. However there may be sometimes a lack of strong correlation between molecular markers and clinical outcomes. The analysis of molecular markers require as well specific infrastructure and highly trained personnel. There is an urgent need to strengthen surveillance systems for anti-malarial drug resistance [42, 43], and develop easy-to-use and low-cost new tools that could provide early warning signals before high levels of resistance to artemisinin and ACT have spread beyond the greater Mekong region.

The aim of this landscape analysis is to provide an overview of the current methods and tools used for surveillance of anti-malarial drug resistance, and to identify current knowledge and technology gaps. The analysis will first provide an overview of existing approaches and methods, including a description of their underlying principles, and then discuss the major advantages and limitations of each approach, concluding finally with suggestions on potential improvements for the different approaches.

## Therapeutic efficacy of anti-malarial drugs

In vivo efficacy assessment consists of prescribing the required dose of anti-malarial drugs (mono- or combination therapies) to patients infected with uncomplicated *Plasmodium* parasites. The WHO has developed and regularly updated methods which has largely contributed to standardize the assessment of anti-malarial efficacy [44]. Having received appropriate treatment, the patients are followed up by parasitological and clinical assessments for a specified number of days (from 28 to 63 days, depending upon the half-life of the medicine assessed), after which the treatment outcome is determined as successful or not. Currently, this assessment is done in routine surveillance and is referred as therapeutic efficacy studies (TES) and have become the gold standard to guide treatment policy in malaria endemic countries [44].

### Therapeutic efficacy studies: history

The procedures for monitoring anti-malarial in vivo drug efficacy were standardized for the first time in 1964 [45] by the WHO after the emergence of *Pf*CQR. The protocol recommended a 7-day observation period. Blood samples were taken and thick films for malaria microscopy were prepared daily during the 7 days of follow-up. The parasitological response to the treatment was classified into sensitive (S) or resistant (R), with three different levels of resistance (Table 1). If the parasitological response was classified as RI, an extended observation for an additional 21 days was usually carried out to distinguish between RI, RII or RIII resistance. The inconvenience of this first protocol included the high workload of taking samples daily during 7 days or more, as well as the fact that the classification of the therapeutic response did not take into account the clinical status of the patient (e.g., persistence of fever, or presence of other malaria symptoms); the protocol was specific for CQ; and the inclusion and exclusion criteria for patient recruitment were not well defined [2]. The short surveillance period of 7 days was also found to result in an underestimation of the true percentage of therapeutic failures, especially for drugs with long half-life. Therefore, this protocol was appropriate for therapeutic efficacy studies in regions of high malaria endemicity, and for the surveillance of drugs with short half-lives.

**Table 1:** Comparison of outcome classifications in therapeutic efficacy study protocols for malaria Adapted from [44–47]

	Year of protocol publication			
	1965	1996	2003	2009
Classifications for treatment efficacy	Sensitivity: clearance of asexual parasitaemia within 7 days of the first day of treatment, *without* subsequent recrudescence	Adequate clinical response (ACR): Absence of parasitaemia on Day 14 irrespective of axillary temperature, without previously meeting any of the criteria of ETF or LTF or Axillary temperature < 37.5 °C irrespective of the presence of parasitaemia, without previously meeting any of the criteria of ETF or LTF	Adequate clinical and parasitological response (ACPR): For low to moderate transmission area: Absence of parasitaemia on day 28 irrespective of axillary temperature without previously meeting any of the criteria of early treatment failure or late clinical failure or late parasitological failure For intense transmission area: Absence of parasitaemia on day 14 irrespective of axillary temperature without previously meeting any of the criteria of early treatment failure or late clinical failure or late parasitological failure	Adequate clinical and parasitological response (ACPR): Absence of parasitaemia on day 28 (or day 42), irrespective of axillary temperature, in patients who did not previously meet any of the criteria of ETF, LCF or LPF
Classifications for treatment failure	Resistance RI: Clearance of asexual parasitaemia as above, but followed by recrudescence before or after day 7	Early treatment failure (ETF): If the patient develops one of the following during the first 3 day of follow-up: Development of danger signs or severe malaria on day 1, 2 or 3, in the presence of parasitaemia; Axillary Temperature ≥ 37.5 °C on day 2 with parasitaemia higher than the day 0 parasite count; Axillary Temperature ≥ 37.5 °C on day 3 in the presence of parasitaemia; Parasitaemia on day 3 ≥ 25% of the parasite count on day 0	Early treatment failure (ETF): For all transmission area: development of danger signs or sever malaria on day 1, day 2, day 3 in the presence of parasitaemia; Parasitaemia on day 2 higher than on day 0 irrespective of axillary temperature; Parasitaemia on day 3 with axillary temperature ≥ 37.5 °C; parasitaemia on day ≥ 25% of count on day 0	Early treatment failure (ETF): Danger signs or severe malaria on day 1, 2 or 3, in the presence of parasitaemia; Parasitaemia on day 2 higher than on day 0, irrespective of axillary temperature; Parasitaemia on day 3 with axillary temperature ≥ 37.5 °C; and parasitaemia on day 3 ≥ 25% of count on day 0
	Resistance RII: Marked reduction of asexual parasitaemia within the first 7 days of follow-up, but no clearance	Late treatment failure (LTF): Development of danger signs or severe malaria in the presence of parasitaemia on any day from day 4 to 14, without previously meeting any of the criteria of ETF; Axillary temperature ≥ 37.5 °C in the presence of parasitaemia on any day from day 4 to 14, without previously meeting any of the criteria of ETF	Late clinical failure (LCF): For low to moderate transmission area: Development of danger signs or severe malaria after day 3 in the presence of parasitaemia without previously meeting any of the criteria of early treatment failure; presence of parasitaemia and axillary temperature ≥ 37.5 °C (or history of fever) on any day from day 4 to day 28 without previously meeting any of the criteria of early treatment failure For intense transmission area: Development of danger signs or severe malaria after day 3 in the presence of parasitaemia without previously meeting any of the criteria of early treatment failure; presence of parasitaemia and axillary temperature ≥ 37.5 °C (or history of fever) on any day from day 4 to day 14 without previously meeting any of the criteria of early treatment failure	Late clinical failure (LCF): Danger signs or severe malaria in the presence of parasitaemia on any day between day 4 and day 28 (or day 42) in patients who did not previously meet any of the criteria of ETF; and presence of parasitaemia on any day between day 4 and day 28 (or day 42) with axillary temperature ≥ 37.5 °C in patients who did not previously meet any of the criteria of ETF
	Resistance RIII: No marked reduction of asexual parasitaemia within the first 7 days of follow-up		Late parasitological failure (LPF): For low to moderate transmission area: Presence of parasitaemia on any day from day 7 to day 28 irrespective of axillary temperature and without previously meeting any of the criteria of early treatment failure or late clinical failure For intense transmission area: Presence of parasitaemia on day 14 and axillary temperature < 37.5 °C without previously meeting any of the criteria of early treatment failure or late clinical failure	Late parasitological failure (LPF): Presence of parasitaemia on any day between day 7 and day 28 (or day 42) with axillary temperature < 37.5 °C in patients who did not previously meet any of the criteria of ETF or LCF

The first major protocol revision was made in 1996, extending the follow up period to 14 days, with blood samples taken only on day 0, day 3, day 7, and day 14 of follow-up. Parasitological and clinical responses were taken into account for the evaluation of therapeutic efficacy [46]. The responses to the treatment were classified into ‘adequate clinical response’ (ACR), ‘early treatment failure’ (ETF) and ‘late treatment failure’ (LTF) (Table 1). However, the protocol remained more appropriate for high transmission regions.

A second major revision was carried out in 2003, with recommendations for the evaluation of drugs in low and moderate transmission settings, to fill the gap of the previous standardized protocol version from 1996 [47]. The protocol included a 28-day follow-up period for low and moderate transmission settings, and a 14-day follow-up period for high transmission settings. A 28-day follow-up period was also recommended for the evaluation of drugs with long half-life. The protocol also suggested the use of genotyping by polymerase chain reaction (PCR) to differentiate recrudescence from re-infections during the follow-up period, especially for studies in high transmission areas [48]. The treatment responses were re-classified into four categories: ‘adequate clinical and parasitological response’ (ACPR), ‘early treatment failure’ (ETF), ‘late clinical failure’ (LCF) and ‘late parasitological failure’ (LPF) (Table 1). Even when this new protocol responded to the needs of different transmission settings, some confusion arose due to the use of the same classification of treatment failures, but with different definitions according to transmission intensity.

### Current protocol

The current protocol was developed in 2009, and incorporates recommendations for all endemic regions in a single procedure (Table 1). For all endemic areas, both clinical and parasitological observations are taken into account for the interpretation of treatment responses [44]. The protocol also includes recommendations for an emergency treatment in case of study exclusions. Patients excluded from the study are either censored or removed from the analysis, depending on the type of analysis that is required, even though, Kaplan–Meier analysis is the preferred to the per protocol analysis [44]. At least 28 days (or 42–63 days for drugs with longer half-lives) are required for follow-up. This protocol includes a recommendation as well to take a blood sample to measure the concentration of the drug at day 7, which is a good predictor of drug absorption and its correlation with treatment failure [49, 50].

### Genotyping to distinguish between recrudescence and reinfection

In the current therapeutic efficacy protocol, systematic genotyping is recommended in cases of clinical or parasitological failure, to distinguish recrudescence from re-infections, using three highly polymorphic genes: merozoite surface protein 1 (*msp1),* merozoite surface protein 2 *(msp2),* and glutamate rich protein (*glurp)* [44]. Genotypic profiles obtained from samples collected on day 0 (i.e., from the initial parasite infection) and on day X (i.e., the day of follow-up observation) are compared. Identical profiles confirm recrudescent cases, while different profiles are indicative of a re-infection. Notably, these genes encode antigens under immune selective pressure, and this might bias the interpretation of dissimilar parasites in paired blood samples [51]. Moreover, despite the WHO recommendation, some studies only use 1 or 2 of the three genes, or use them sequentially and only test some of the genes, and other use more than three genes, which limits the comparison between different studies and could compromise the interpretation of the results [52]. The results of the genotyping are often detected by agarose gel, and there can be high inter-individual variation in results interpretation depending on the quality of the gel and the experience of the laboratory technician [53]. This is due to the lack of resolution of the current genotyping methods that do not allow to correctly distinguish polymorphisms on agarose gel for digested PCR products with long fragments such as those from *glurp* [54]. Moreover, genotyping could not give accurate estimates, especially in area of high transmission intensity due to the high number of infecting parasite strains (multiplicity of infection) [52].

Other methods have been developed to distinguish recrudescent and new infections, using microsatellite markers [55] and capillary electrophoresis [56, 57]. Microsatellites are simple sequence repeats in the *Plasmodium* genome, generally not more than three nucleotides, and hundreds have been described [58]. They are generally not under immune selection, and the sizes of alleles fall at predictable, discrete lengths that may enable easy comparison across multiple samples and laboratories [59]. By measuring the size of microsatellites using capillary electrophoresis, which has a resolution of one nucleotide and is highly reproducible, the full diversity of length polymorphisms present in a population can be evaluated [55]. Many procedures for genotyping by microsatellites have been discussed [60], and some studies have shown that it is difficult to conclude with certainty the distinction between a recrudescence and a reinfection. The use of microsatellite markers in combination with *msp1*, *msp2*, and *glurp* genes may improve the sensitivity and standardization methods of *P. falciparum* genotyping [55, 61, 62].

### Pharmacokinetics (PK)/pharmacodynamics (PD)

It has been shown that host factors, such as bioavailability and immune response, can influence the therapeutic response [63, 64]. Apparent drug failures may in fact reflect issues with the metabolism of the drug rather than innate parasite resistance. The impact of drug metabolism on the treatment outcome can be assessed by PK (dynamics of the drug concentration resulting from administration of a certain drug dose) and PD (impact of a certain drug concentration on the parasite density) [65]. Bioavailability is one of the most important factor, referring to the proportion of the absorbed drug that is transformed into the active metabolite, enters the blood circulation, and has an anti-malarial effect [49, 65]. Only a few years ago, the WHO started promoting pharmacology assessment as part of anti-malarial efficacy studies, by publishing specific methods and procedures [50]. The WHO’s protocol recommends taking a blood sample (venous or capillary blood) from the patient, several times per day, to assess drug concentration dynamics over the course of the treatment. These data are analysed using different PK models appropriate for each drug [66]. The results show if the drug has reached the required therapeutic concentration that correlates with elimination of the parasite, or if the concentration has been sub-optimal, in which case a treatment failure should not be interpreted as parasite resistance [66, 67]. Studies with different anti-malarial drugs have shown that in some patients, especially young children, who are frequently those involved in therapeutic efficacy studies in high transmission settings, tend to receive a lower dose of the drug leading to suboptimal blood concentrations [68, 69]. This may lead to patient outcomes misclassifications as treatment failure due to drug resistance whereas the patient didn’t reach the optimal drug concentration to clear the parasites.

## In vitro and ex vivo phenotypic assay for anti-malarial susceptibility assessment

### Principle

**Fig. 1:**
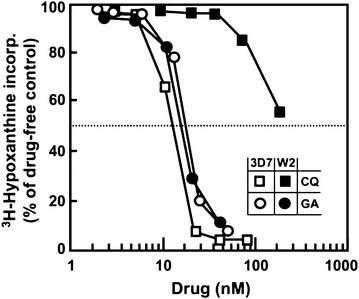
Inhibition of CQ-sensitive (3D7) and CQ-resistant (W2) P*. falciparum* by geldanamycin (GA) and chloroquine (CQ) (*Source* [72])

The assessment of *P. falciparum* parasites susceptibility to anti-malarial drugs can be performed phenotypically, using parasite strains collected from patients (ex vivo) or with culture-adapted isolates (in vitro) [70]. The assessment can be done by culturing parasites in the presence of anti-malarial drugs at varying concentrations to determine the growth inhibitory effect of the drugs (Fig. 1), or by exposing parasites to a specific high concentration for a relatively short period [71]. The parasite culture is done in laboratory flasks with liquid medium and red blood cells, supplemented with amino acids, human serum, or bovine serum albumin (BSA). Antibiotics can also be added to the culture medium to avoid bacterial contamination. The susceptibility assays are usually done in assay plates (96-, 24-, or 12-well) which have been previously coated with varying concentrations of the anti-malarial drugs. The parasite growth is then measured using various techniques, and results used to determine either the concentration that inhibits parasite growth by 50% (50% inhibitory concentration; IC_50_) [73] or the survival rate [71]. Currently, in vitro*/*ex vivo drug sensitivity assays use one of several different methods to measure parasite growth [74], as described below and in Table 2.

**Table 2:** Laboratory methods to assess in vitro/ex vivo susceptibility of Plasmodium falciparum parasites to antimalarial drugs

	WHO microtest	Isotopic	ELISA	Flow cytometry	SYBR green	RSA
Infrastructure	Biosafety laboratory level 2 for parasite culture	Biosafety laboratory level 2 for parasite culture	Biosafety laboratory level 2 for parasite culture	Biosafety laboratory level 2 for parasite culture	Biosafety laboratory level 2 for parasite culture	Biosafety laboratory level 2 for parasite culture
Equipment	Refrigerator Freezer Microscopy Biosafety cabinet Incubator with gas (CO_2_, O_2_ and N_2_)	Refrigerator Freezer Microscopy Biosafety cabinet Incubator (CO_2_ and O_2_) Liquid scintillation counter	Refrigerator Freezer Microscopy Biosafety cabinet Incubator with gas (CO_2_, O_2_ and N_2_) Spectrophotometer	Refrigerator Freezer Microscopy Biosafety cabinet Incubator with gas (CO_2_, O_2_ and N_2_) Flow cytometer	Refrigerator Freezer Microscopy Biosafety cabinet Incubator with gas (CO_2_, O_2_ and N_2_) Fluorimeter	Refrigerator Freezer Microscopy Biosafety cabinet Incubator with gas (CO_2_, O_2_ and N_2_) Flow cytometer)
Reagents	Reagents for culture	Reagents for culture [^3^H] hypoxanthine	Reagents for culture HRP2/pLDH monoclonal antibodies Reagents for ELISA	Reagents for culture Fluorochrome	Reagents for culture SYBR Green	Reagents for culture Fluorochrome
Incubation time (h)	24–30	48	72	48	48	48
Time to results (12 samples) (h)	38–42	52	80	54	52	60
Reproducibility	Variable	Good	Variable	Good	Good	Variable to good
Cost by sample (USD $)	≥ 5	≥ 5	1–5 (HRP2) 0.5–2 (pLDH)	≥ 5	0.08	≥ 5
Advantages	No heavy equipment	Automatic reading	Relatively inexpensive	Highly sensitive	Inexpensive Short Procedure	No heavy equipment (except if using flow cytometry)
Disadvantages	Labour intensive Requires quality assured microscopy Difficult to standardize Expensive	Use of radioactive reagents Heavy equipment Expensive	High inter-variability between laboratories and users No commercially available Kit for pLDH	Heavy equipment Expensive	Underestimation of parasitemia because of DNA-binding proteins competing with the dye Heavy equipment Interaction between drugs and the dye	Labour intensive (microscopy) Requires quality assured microscopy Difficult to standardize (microscopy) Heavy equipment (if using flow cytometry) Expensive

### Microscopy (WHO microtest)

The principle of the assay is based on counting parasite growth by microscopy. Parasites are cultured with different concentrations of drugs for 24–30 h. The baseline parasitemia differs between the different techniques, and varies from 0.1 to 1% of red blood cells being infected. Parasite growth in the different plate wells with different drug concentrations is determined by counting the parasites by microscopy, on a thin film, after Giemsa staining [75, 76]. One must take into account the number of parasites at different developmental stages (ring stage or schizont) for the interpretation of the results.

### Isotopic test

The principle of the assay is based on measuring parasite growth by adding radioactive dye in the culture that is incorporate into parasite DNA. Parasites are cultured with different concentrations of a drug for 48 h, and a radioactive marker, i.e., tritium labelled hypoxanthine, is added to the culture medium [77, 78]. Hypoxanthine is a DNA precursor, and the tritium-labelled hypoxanthine is incorporated into the parasite DNA during the culture phase. After the 48 h incubation period, the culture is filtered through filter paper and the paper dried. A scintillating liquid is added to the paper, and in a beta-counter machine, the parasite DNA is measured as counts per minute (CPM), and resulting values are used to calculate the IC_50_.

### ELISA (HRP2 and pLDH)

The principle of the assay is based on assessing parasite growth by measuring the concentration of proteins produced by the cultured parasites [79]. Parasites are cultured with different concentrations of a drug for 72 h. After 72 h, the culture plates are frozen at − 20 °C and thawed (several times if needed) to ensure cell lysis. In parallel, ELISA plates are coated with monoclonal antibodies directed against the *Plasmodium spp.* LDH (lactate dehydrogenase), or the *P. falciparum*-specific HRP2 (histidine-rich protein 2) at 4 °C overnight [80, 81]. The wells of the antibody coated plates are then incubated with parasite culture mixes transferred from the thawed parasite culture plates. After an incubation period of 2 h, biotinylated antibodies and colorimetric detection reagents are added. Finally, the plates are read on a spectrophotometer at 450 nm, and resulting absorbance values are used to calculate the IC_50_ and determine the parasite susceptibility to anti-malarial drugs [82, 83].

### Fluorescent markers

The principle of the assay is based on measuring parasite growth using a fluorescent marker that will react with DNA and RNA [84]. Parasites are cultured with different concentrations of a drug for 48 h or 72 h. After 48/72 h of incubation, the parasite culture plate is frozen at − 80 °C until the SYBR Green I assay is performed. The plate is thawed for 2 h at room temperature on the day of analysis, and the contents are shaken briefly, before transferring to a new plate, where SYBR Green is added and incubated at room temperature for 1 h. Finally, the plate is read on a fluorimeter, the intensity of fluorescence corresponding to the quantity of the DNA in the culture, and the IC_50_ is determined from the obtained values.

### Flow cytometry

The principle of the assay is based on measuring parasite growth by counting the number of infected red blood cells. Parasites are cultured for 48 h with different concentrations of a drug [85]. The method is based on the detection of infected red blood cells by marking intra-erythrocytic parasite DNA with a fluorescent dye. Various types of permeable markers can be used to mark the DNA [85]. The mixture of infected and non-infected red blood cells is then analysed in a cytometer, and the results analysed to determine the amount of infected cells, hence the number of parasites having grown in absence or in presence of the anti-malarial drug, to determine the IC_50_ [86, 87].

### Ring stage survival assay (RSA)

This method was developed specifically to assess resistance of *P. falciparum* parasites to artemisinin derivatives that cannot be well detected by the classical in vitro*/*ex vivo assays [71, 88]. This is due to the fact that the decreased susceptibility to artemisinin affects the ring stages only [89], hence a resistance phenomenon cannot be well detected in a culture that goes through all the parasites stages. For in vitro assays, parasites are cultured without any drug to reach a high parasite density (≥ 0.2%), then a tight synchronization step (ring-stages aged from 0 to 3 h) is performed to eliminate the schizont stages, and the ring stage parasites are placed into culture in the presence of 700 nM of dihydroartemisinin (DHA). This concentration represents the typical therapeutic concentration found in patients treated with artemisinin derivatives. DHA is removed after 6 h (a physiologically relevant duration), and the parasites are placed into a fresh culture mix for another 66 h. For ex vivo assays, samples collected from patients are processed within 24 h. Plasma is removed and the blood washed three times in RPMI-1640. If the parasitemia is greater than 1%, it is adjusted to 1% by adding uninfected erythrocytes, but parasites are not experimentally synchronized, they are directly exposed to 700 nM of dihydroartemisinin (DHA) for 6 h, and then placed into a fresh culture mix for another 66 h. For both in vitro and ex vivo, survival rates are assessed microscopically or by flow cytometry by counting the proportion of viable parasites that developed into second-generation rings or trophozoites with normal morphology at 66 after drug removal [90].

## Molecular methods for the detection of genetic polymorphisms associated with anti-malarial drug resistance

### Principle

Molecular methods have allowed for a better understanding of the emergence and spread of anti-malarial drug resistance. In the last two decades, the mechanisms of resistance to the most widely used anti-malarial drugs have been revealed in part using molecular techniques, and anti-malarial resistance is often associated with single nucleotide polymorphisms (SNPs) or amplifications of the genes coding for drug target proteins or transporters (CNVs) [91]. Various methods have been developed for the assessment of these known resistance markers. Resistance to chloroquine is associated with point mutations in two different genes: *P. falciparum* chloroquine-resistance transporter (*Pfcrt*) and *P. falciparum* multidrug resistance gene 1 (*Pfmdr1*) [92–94]. Resistances to sulfadoxine/pyrimethamine are associated with point mutations in *P. falciparum* genes coding for dihydrofolate reductase (*Pfdhfr*) [95, 96] and dihydropteroate synthetase (*Pfdhps*) [97], while resistance to mefloquine has been associated with gene amplification in *Pfmdr1*gene [98, 99]. Mutations in the parasite *cytochrome b* are associated with atovaquone resistance [100] and resistance to piperaquine has been associated with gene amplification in *plasmepsin 2* and *3* genes [101, 102]. More recently, decreased susceptibility to artemisinin derivatives has been associated with point mutations in the propeller domain of a Kelch gene located on the chromosome 13 (K13) [33].

The basic principle of most of methodologies assessing genomic markers associated with drug resistance is based, after DNA extraction, on the amplification of the gene or loci of interest, using the polymerase chain reaction (PCR). To increase the detection sensitivity, a second amplification can be used to amplify the PCR product of the primary PCR (nested PCR). In resource-limited settings, blood samples are often collected and dried onto filter papers and then stored and transported appropriately to the laboratory. Parasite DNA obtained from dried blood spots can be stable when adequately stored until analysis (several years at room temperature). The transport of these biological samples therefore doesn’t require any specialized equipment for storage; however it is important that the blood spots are well dried to avoid growth of fungi and deterioration of the DNA. It is also critical to avoid cross contamination between different samples when storing and cutting the blood spots. The DNA extraction can be performed using a variety of methods, ranging from simple Chelex methods to commercial kits [103]. The amplification by PCR only requires a small amount of extracted DNA.

### Restriction fragment length polymorphism (RFLP)

The PCR products are digested with specific restriction enzymes, to determine if the sequence of the codons of interest is present [104]. These restriction enzymes only cut the nucleotide sequence at specific sites which results in PCR product fragments of specific sizes. After enzymatic digestion the digested amplification products are then analysed by agarose gel electrophoresis to determine the length of the digested DNA products. The result can then be interpreted as ‘mutant’ (i.e., mutation associated with drug resistance), ‘wild-type’ (i.e., absence of mutation associated with drug sensitivity) or mixed (presence of both mutant and wild-type alleles, i.e. a mixture of parasite strains) [105].

### Sanger sequencing (capillary electrophoresis)

Sequencing can be performed on the gene or loci of interest. Sanger sequencing (chain termination sequencing) is a method of DNA sequencing based upon the selective incorporation of chain terminating dideoxynucleotides (ddNTPs) during in vitro DNA replication [106]. There are two classical methods of Sanger sequencing which utilize fluorescently labelled primer or labelled dNTPs. In most cases both strands of the targeted genomic DNA are sequenced. The sequences are then reassembled using dedicated software and compared to a sequence from reference strain (often 3D7) to look for new point mutations. The length of the DNA target which can be sequenced with high confidence is based upon the technique and the method utilized and how well the method has been optimized. In general 400–700 bases is an average read length for Sanger sequencing.

### Next generation sequencing

Next generation sequencing (NGS) can be performed on the gene or loci of interest or the whole genome to look for potential new mutations or mechanisms of resistance. NGS can be used as well to track the origin and spread of resistant parasites using microsatellites data analysis [36, 37, 107]. Different samples are pooled either after or before DNA extraction and are sequenced together. A DNA library is then prepared consisting of small fragments of the DNA, not all of the same size, but on average 100 base pairs. NGS systems are able to sequence millions of small base pairs in parallel based on the “shotgun” approach in which millions of short nucleotides are sequenced in parallel for only one strand of the DNA. Deep sequencing refers to sequencing a genomic region multiple times, sometimes hundreds or even thousands of times. This NGS approach allows detection of rare clonal types compared to the classic sequencing by capillary electrophoresis [108]. The sequencing data are analysed by bioinformatics tools to reconstruct the gene of interest or the whole genome and compare it to a reference strain (usually 3D7) [109].

### Real-time-PCR

Real-time PCR can be used to detect both SNPs and gene copy number. Real-time PCR is a PCR conducted on a specific piece of equipment, which allows for the real time observation of the DNA amplification using nucleic acid stain dyes (i.e. SYBR green), fluorescence-labelled amplification primers and/or probes. When the technique is used for quantitative assay to detect changes in gene copy number, fluorescent or intercalating dyes are added to the PCR mix to detect PCR product as it accumulates in real time during PCR cycles. The measured fluorescence is proportional to the total amount of amplicon; the change in fluorescence over time is used to calculate the amount of amplicon produced in each cycle. The cycle number at which the fluorescent signal emitted during the amplification first cross a threshold (Ct) corresponds the amount of target that was present in the reaction at cycle 1 of the reaction (starting concentration) [110]. In order to perform SNP genotyping, two specific probes labelled with different dyes are used, the first for the wild type allele and the second for the mutant allele. Those oligonucleotides are constructed with a fluorescent dye attached at the 5′ end and a quencher at the 3′ end are used to detect SNPs. When the specific target sequence is present, the oligonucleotide anneals and during the extension phase of PCR, the probe is cleaved. The cleavage of the probe will remove the probe from the target DNA strand and separates the reporter dye from the quencher, allowing the increase of the fluorescence signal which can be detected. This process is repeated each cycle, resulting in an increase in fluorescence intensity proportional to the amount of the PCR product. The mutant, wild-type, or mixed genotype can therefore be determined by observing the real-time increase of the fluorescence of the given fluorophore(s) [111].

### Ligase detection reaction fluorescent microsphere (LDR-FM) assay

PCR products are amplified in a multiplex ligase detection reaction containing specific primers for each mutation and common primers. The specific primers are designed in a way that they contain a tag at the 5′ end complementing a sequence attached to a Luminex-Tag bead and 3′ end specific for the mutation of interest. After this second amplification, products are hybridized to Luminex-Tag beads. To quantify the abundance of different alleles, labelled products are then run on a Luminex instrument, and results are read as fluorescence intensity for each reaction in a 96-well format [112].

### New molecular methods in development

In addition to the most common methods described above, new molecular methods have been developed over the last years or are being developed. The first step of these methods is the same as for the ones above, consisting of a PCR amplification. However there are different approaches for the subsequent detection of the mutations.

### Nucleic acid lateral flow immunoassay (NALFIA)

For this technique, there is no DNA extraction step needed. The blood is directly added to the PCR reaction, and the target gene or part of gene is amplified for 1 h. Thereafter, the product can be visualized with NALFIA, which is a rapid immunochromatographic test to detect labelled amplicon products on a nitrocellulose stick coated with specific antibodies. The amplicons are labelled via specific primers that contain a biotin molecule and a hapten. This complex is detected by direct interaction with a colloidal, neutravidin-labelled carbon particle [113]. Like a malaria rapid diagnostic test, the NALFIA has a positive control, which is the human housekeeping gene glyceraldehyde 3-phosphate dehydrogenase (GAPDH). This technique has already been used to detect molecular markers associated with anti-malarial drug resistance and showed good correlation with standard sequencing and real-time PCR methods [113].

### Q-poc™

This method has been developed by a company called Quantum DX in collaboration with academic institutions [114]. The Q-POC™ is a simple handheld molecular device that could provide results in less than 20 min. The device does include a cassette to both collect the sample and perform sample preparation, DNA amplification and detection by microarray. The device can differentiate species of *Plasmodium* parasites, and can also detect the different molecular markers associated with anti-malarial drug resistance. The company in collaboration with St George’s, University of London, is developing a malaria specific assay for molecular markers of anti-malarial resistance under the EU funded project NanoMal [114].

## Discussion and outlook

The current decrease of *P. falciparum* sensitivity to artemisinin derivatives and the development of resistance to partner drugs in Southeast Asia could jeopardize the gains made over the last decade in the fight against malaria [32]. To delay the spread of drug-resistant parasite strains, simplified, standardized and structured surveillance system are needed [42, 115, 116] to detect spatial and temporal trends at best in real time [117, 118]. Each of the three approaches used to assess anti-malarial drug resistance, i.e. in vivo, in vitro, and molecular assays, has its own advantages and disadvantages (Table 3), however their combination in a standardized and well-coordinated way could substantially not only improve the results, but also decrease the required efforts (labour and financial) for anti-malarial drug resistance surveillance.

**Table 3:** Advantages and disadvantages of the different approaches for monitoring anti-malarial resistance

	Advantages	Disadvantages
In vivo	Relatively easy to standardize No heavy equipment required Provides results directly obtained from patients Provides the evidence required for modifying treatment policies Helps to define the first line and second line treatment for case management Can provide required safety data Confirms association of parasite resistance with in vitro test results (IC_50_ values) or molecular resistance markers	Logistics constraints (long follow-up with many patients lost to follow up, lack of patients in low transmission settings, expensive) Potential over-estimation of treatment failures because of: inter-individual variation in pharmacokinetics including poor absorption, rapid elimination (diarrhoea, vomiting) and/or insufficient or poor biotransformation of pro-drugs because of human genetic characteristics; extrinsic factors such as poor patient compliance (if the totality of treatment is provided), incorrect dosage, poor drug quality or poor microscopy Potential under-estimation of treatment failures because of host factors such as the immunity or poor microscopy
In vitro	Provides the intrinsic parasite susceptibility to the drug without confounding factors such as immunity and pharmacology	Difficult to standardize Require a special design (concentration and duration) for certain drugs (i.e. RSA, PSA) Requires good infrastructure and highly trained staff Results not always associated with therapeutic efficacy
Molecular	Provides direct information on the resistance status of the parasite. When they are validated, their prevalence in a parasite population are often a good indicator of the level of clinical resistance Can provide useful information on the spread of resistance Relatively easy to implement	Requires good infrastructure and highly trained staff Results not always associated with therapeutic efficacy

The in vivo approach has a standardized protocol from WHO [44], and remains the gold standard to evaluate the therapeutic efficacy of anti-malarial drugs. However, in vivo studies have huge logistical and financial constraints [41], and several limitations, such the quality of microscopy. Standardized methodology to perform good quality microscopy with suggestions for microscopist selection, training, and continuous evaluation, including internal and external quality control schemes should be used to improve the quality of microscopy [119]. Digital microscopy could be an alternative, removing the visual inspection of the slide that is error-prone and time consuming [120]. Field testing have shown that digital microscopes can have sensitivity comparable to PCR [121, 122], however, complex algorithms need to be developed to analyse the data, and large scale evaluation in field settings are required to assess their specificity and sensitivity compared the current gold standard, quality assured optic microscopy [120]. Another limitation is the interpretation of the genotyping results to distinguish recrudescence from reinfection. Indeed, most of TES are using *msps1/msp2/glurp* genotyping, as recommended by the WHO protocol [44]. There is high variability in results interpretation based on agarose gel quality; and different studies are using different markers, which makes difficult to compare PCR-corrected treatment outcomes [123]. Moreover, the systematic use of *glurp* in conjunction with *msp1* and *msp2* could lead to misclassification of treatment outcomes due to the non-detection of minority strains in co-infections in high endemic settings [54]. The sensitivity of genotyping could be improved by using capillary electrophoresis [56, 57], and the use of microsatellites markers [55, 124].

The use of in vitro/ex vivo techniques require good infrastructure for parasite culture [125], and is further hampered by the difficulty to compare results across different assay methods [126], because of the high variability in results between methods and across different laboratories [125]. Moreover, it is difficult to standardize protocols for parasite culture, as there is always high intra-assay variability in parasite growth whatever protocol is used [127]. In vitro/ex vivo techniques are more appropriate for national reference laboratories, where they could be used for monitoring parasite susceptibility for drugs for which no validated molecular markers are available [128, 129], using per example a sensitive and cheap technique such as SYBR Green [130]. The method gives IC50 results comparable to HRP2 ELISA, and is more reproducible than ELISA methods [131]. However, the technique has its own limitations such as interference with some small proteins that could compete with the dye to bind the DNA [132], or drug interference with the dye that could cause high background [133]. The development of standardized analytical tools such as the In vitro Analysis and Reporting tool (IVART), a high throughput in vitro*/*ex vivo data analysis tool that can analyse data from different in vitro*/*ex vivo assays could help in standardizing analytical methodologies [73].

Blood sample collection and DNA extraction are crucial steps in the assessment of the prevalence of anti-malarial drug resistance molecular markers, and have shown to have a substantial impact on the PCR product [134]. Long storage of dried blood spots should be done at − 20 °C as opposed to current practices of storing DBS at room temperature and DNA extraction methods should be selected appropriately [134]. Even though simple, the RFLP method may imply logistical and financial constraints for laboratories to implement, in addition to the high workload required [112]. Furthermore the method can also present problems of quality and reproducibility if not used with the required rigour (Additional file 1), cannot detect minority strains and gene copy number changes [135]. Real-time PCR, and sequencing have the advantage of their higher throughput, their increasing availability and decreasing cost [108, 111]. For sequencing, a consortium approach could be used to gather data from different settings on a common platform [136, 137]. Regional reference laboratories with sequencing capacity could be established as well, allowing multiple countries to share the cost burden, and this could be based on previous experiences in developing regional networks for anti-malarial resistance monitoring [138, 139]. Indeed, the cost of sequencing has decreased substantially over the last decade, and laboratories in developing countries are acquiring the expertise in sequencing and have the required equipment and qualified staff. Nevertheless some investment would be needed to develop data analysis capacity by training local biostatisticians. New technologies are also being developed and could simplify and decrease the costs of anti-malarial drug resistance surveillance [113, 114].

Various initiatives aiming at standardizing methodologies for the surveillance of anti-malarial drug resistance have been implemented. Regional networks such as the East African Network for Monitoring of Antimalarial Treatment (EANMAT) and The Amazon Network for the Surveillance of Antimalarial Drug Resistance (RAVREDA) were created in 1997 and 2001, respectively, to monitor the spread of anti-malarial drug resistance [138–140]. While RAVREDA is still operating with support from PAHO and USAID, EANMAT is no longer active since 2006. A global network has been established in 2009, as the Worldwide Antimalarial Resistance Network (WWARN) for the surveillance of anti-malarial resistance globally [117]. This is mainly a network of researcher institutions aimed at providing up to date spatial and temporal information on anti-malarial resistance on a global level. The PMI supported anti-malarial resistance network (PARMA) is another initiative aiming at standardizing, data sharing and capacity building in African PMI-funded countries [141]. However, efforts to bring together the scientific community and national stakeholders, especially the National Malaria Control Programmes (NMCPs) remain challenging. The experience from all these different initiatives show that it is difficult to effectively coordinate all the different stakeholders with a common agenda focused on surveillance of anti-malarial drug resistance. More importantly, these networks strongly depend on donor funding, which if stopped, negatively affect the survival of these initiatives as was the case for EANMAT. Fortunately RAVREDA and PARMA are still active with support from USAID/PMI, while WWARN continues to operate as part of a larger platform, the Infectious Diseases Data Observatory (IDDO) with financial support from various donors. Developing and maintaining those networks are relatively costly and highly demanding in terms of staff and time. Moreover it is difficult to develop guidelines, protocols and standardized methodologies that will respond to the needs and opinions of all involved stakeholders.

The development of new, user-friendly and affordable molecular methods for anti-malarial resistance surveillance could provide useful real-time information on spatial and temporal trends for anti-malarial drug resistance to monitor the appearance and spread of anti-malarial resistance. Moreover, simplified sample processing would decrease errors and improve the final data quality. Combined with a good external quality assessment system such as a proficiency testing program; this could help to improve the standard quality of data. Frequent cross sectional surveys and longitudinal studies at sentinel sites using molecular markers could be used as early warning signals [63, 64]. The advent of affordable new molecular tools, such as next generation sequencing, could substantially improve the information provided by molecular markers of resistance in combination with microsatellites markers, and would allow not only to assess the prevalence of those markers, but also how they spread, allowing to make predictions on the future spread of resistance. In parallel, cheaper and robust equipment as well as simplified on-line data analysis tools need to be developed to allow the analysis of the high amount of data obtained from sequencing by staff with limited training from malaria endemic countries.

## Conclusion

The development of new molecular methods for detecting SNPs or CNVs associated with anti-malarial drug resistance, combined with the continuous support of networks including national, regional reference laboratories in malaria endemic countries all participating in a similar proficiency testing programme, and/or global efforts such as WWARN could help facilitating and sustaining anti-malarial drug resistance surveillance. These new tools will complement effectively in vivo data, but would require external evaluation scheme through proficiency testing programmes to ensure data quality and data standardization and collation of information to provide a comprehensive picture of anti-malarial resistance to guide policy.

## Additional file

**Additional file 1: Table S1.**Molecular methods comparative table. **Table S2.** Detection methods for molecular assays.

## Abbreviations

ACR: adequate clinical response
ACPR: adequate clinical and parasitological response
ACT: Artemisinin-based Combination Therapy
CQ: chloroquine
CQR: chloroquine resistance
DHA: dihydroartemisinin
ddNTP: dideoxynucleotide
DNA: deoxyribonucleic acid
dNTP: deoxynucleotide
EANMAT: East African Network for Monitoring Antimalarial Treatment
ELISA: enzyme-linked immunosorbent assay
ETF: early treatment failure
GAPDH: glyceraldehyde 3-phosphate dehydrogenase
GLURP: glutamate rich protein
HRP2: histidine-rich protein 2
IDDO: Infectious Diseases Data Observatory
LDH: lactate dehydrogenase
LDR-FM: ligase detection reaction fluorescent microsphere
LTF: late treatment failure
LPF: late parasitological failure
MSP1: merozoite surface protein 1
MSP2: merozoite surface protein 2
MQ: mefloquine
NALFIA: nucleic acid lateral flow immunoassay
NGS: next generation sequencing
NMCP: National Malaria Control Programme
PCR: polymerase chain reaction
PD: pharmacodynamics
PFCRT: *Plasmodium falciparum* chloroquine-resistance transporter
PFDHFR: *Plasmodium falciparum* dihydrofolate reductase
PFDHPS: *Plasmodium falciparum* dihydropteorate synthetase
PK: pharmacokinetics
PFMDR 1: *Plasmodium falciparum* multidrug resistance 1
PMI: President’s Malaria Initiative
PvCQR: *Plasmodium* vivax resistance to chloroquine
R: resistant
RAVREDA: Amazon Network for the Surveillance of Antimalarial Drug Resistance
RNA: ribonucleic acid
RSA: ring stage survival assay
S: sensitive
SNP: single nucleotide polymorphism
SP: sulfadoxine-pyrimethamine
USAID: United States Agency for International Development
WHO: World Health Organization
WWARN: Worldwide Antimalarial Resistance Network
