# Supplementary material for: Tools for surveillance of anti-malarial drug resistance: an assessment of the current landscape
Source: Malar J. 2018 Feb 8;17:75. doi: 10.1186/s12936-018-2185-9 (PMC5806256; doi:10.1186/s12936-018-2185-9)
Supplement: Supplementary file 1 — Additional file 1: Table S1.Molecular methods comparative table. Table S2. Detection methods for molecular assays. [file 12936_2018_2185_MOESM1_ESM.docx]

**Supplementary files**

**Table 1: Molecular methods comparative table**

|  | RFLP | Sequencing (Sanger) | Next Generation Sequencing: NGS | RT-PCR |
| --- | --- | --- | --- | --- |
| Advantages | - Requires less equipment (thermocycler, UV gel workstation) - Relative low cost (thermal cyclers, gel electrophoresis) - Equipment less demanding in terms of maintenance | - Adapted for analysis of multiple samples and genes/SNPs of interest - Equipment might be available in the laboratory for other activities (standard sequencing) - Useful for the discovery of new mutations - Useful for genome wide associated studies (GWAS) - Sequencing of long contiguous fragments of DNA (> 500 nucleotides) - Used to confirm results of the NGS sequencing results | - High throughput - Adapted for analysis of multiple samples and genes/SNPs of interest - Useful for the discovery of new mutations - Useful for genome wide associated studies (GWAS) - Can sequence several samples at a time - Can detect minority strains | - Equipment might be available in the laboratory for other activities (RT-PCR for other studies) - Less workload : low number of manipulation steps, only a single amplification step - Mixed infections with both wild type and mutant genotypes can be detected with greater sensitivity than agarose gel-based methods - Need of less working rooms as compared to the other methods   Less time required for analysis (2 hours for simultaneous amplification and detection)   - Nearly no risks of contamination (closed system) - Adapted for simultaneous analysis of multiple samples (use of 96 well plates) |
| Disadvantages | - High workload - High risks of post-PCR contamination - Sometimes confusing interpretation in case of mixed genotypes (wild-type and mutant) - Not adapted for the analysis of large sample numbers and multiple genes and SNPs of interest | Multiple steps involving high workload  Need of specific and expensive equipment,  and reagents  Maintenance and repair costs can be expensive  Can only sequence one sample at a time  Cannot detect minority strains | Need of specific and expensive equipment, and reagents  Multiple steps involving high workload  Maintenance and repair costs can be expensive  Higher error rate in sequencing compared to classic sanger method  Require a high expertise in bioinformatics   - Only sequencing of short fragments | - Need of specific and expensive equipment, - Requires specific and expensive reagents - Maintenance repair costs can be expensive. |

**Table 2: Detection methods for molecular assays**

|  | Fluorescent probes | Dot blot | Ligase detection reaction fluorescent microsphere (LDR-FM) assay |
| --- | --- | --- | --- |
| Advantages | - Low workload - Easy to implement | - Low workload - Easy to implement | - Low workload - Easy to implement |
| Disadvantages | - Rarely used - Not cost-effective compared to sequencing or RT PCR | - Rarely used - Low specificity - Not cost-effective compared to Sequencing or RT PCR | - Require a Luminex machine and tags that are expensive |
